# Mutations in *Ehrlichia chaffeensis* Causing Polar Effects in Gene Expression and Differential Host Specificities

**DOI:** 10.1371/journal.pone.0132657

**Published:** 2015-07-17

**Authors:** Chuanmin Cheng, Arathy D. S. Nair, Deborah C. Jaworski, Roman R. Ganta

**Affiliations:** 1 Center of Excellence for Vector-Borne Diseases, Department of Diagnostic Medicine/Pathobiology, College of Veterinary Medicine, Kansas State University, Manhattan, KS 66506, United States of America; 2 Department of Entomology and Plant Pathology, Oklahoma State University, Noble Research Center, Stillwater, OK 74074, United States of America; The Johns Hopkins University School of Medicine, UNITED STATES

## Abstract

*Ehrlichia chaffeensis*, a tick-borne rickettsial, is responsible for human monocytic ehrlichiosis. In this study, we assessed *E*. *chaffeensis* insertion mutations impacting the transcription of genes near the insertion sites. We presented evidence that the mutations within the *E*. *chaffeensis* genome at four genomic locations cause polar effects in altering gene expressions. We also reported mutations causing attenuated growth in deer (the pathogen’s reservoir host) and in dog (an incidental host), but not in its tick vector, *Amblyomma americanum*. This is the first study documenting insertion mutations in *E*. *chaffeensis* that cause polar effects in altering gene expression from the genes located upstream and downstream to insertion sites and the differential requirements of functionally active genes of the pathogen for its persistence in vertebrate and tick hosts. This study is important in furthering our knowledge on *E*. *chaffeensis* pathogenesis.

## Introduction

*Ehrlichia chaffeensis* is an *Amblyomma americanum* tick transmitted rickettsial pathogen causing persistent infections in people and several other vertebrates [1–3]. White-tailed deer is the reservoir host for *E*. *chaffeensis*, while humans, dogs and other vertebrate hosts, such as coyotes and goats, are regarded as incidental hosts [4–7]. People with human monocytic ehrlichiosis (HME) caused by *E*. *chaffeensis* may exhibit flu like symptoms leading to significant morbidity and the disease can be fatal if untreated, particularly in people having compromised immunity [3,8]. People undergoing blood transfusions and organ transplantations are also at high risk in acquiring HME [9,10]. The limited therapeutic option to a single antibiotic class and the non-availability of vaccines are the added challenges [11]. Deer, dog and *A*. *americanum* tick infection studies are ideal for mapping genes essential to *E*. *chaffeensis* growth and persistence as they are the recognized reservoir host, an incidental host and the tick vector, respectively [12]. Canine host is an ideal incidental host model similar to humans in acquiring *E*. *chaffeensis* infections from *A*. *americanum* ticks [13]. *E*. *chaffeensis* infections in deer and dog are very similar in exhibiting clinical symptoms and pathogen persistence [12].

Recently, we described several transposon insertion mutations in *E*. *chaffeensis* [14]. We also described that mutations causing transcriptional inactivation of three putative membrane protein genes cause rapid clearance of the organism from deer (reservoir host) [14]. Here, we investigated the impact of transposon mutations on the pathogen’s growth in an incidental host (dog) and in its tick vector. We also investigated the impact of mutations in altering transcriptional activities of genes near to the insertion sites.

## Materials and Methods

### *In vitro* cultivation of *E*. *chaffeensis*

*E*. *chaffeensis* Arkansas isolate, wild-type and the mutants, were continuously cultivated in the canine macrophage like cell line, DH82 [15].

### Clonal purification and verification of *E*. *chaffeensis* mutants

Transposon mutants of *E*. *chaffeensis* [14] were clonally purified by the limiting dilution technique [16]. Briefly, host cell-free *E*. *chaffeensis* mutant pools were prepared [14], the numbers of organisms were estimated using a hemocytometer, and about one bacterium was transferred per chamber to a 48-well plate containing confluent DH82 cells and incubated at 37°C with 5% CO_2_. When the infectivity reached to about 80%, 0.7 ml culture from each well was harvested for genomic DNA isolation [14]. The remaining culture was then transferred to a T25 flask containing confluent DH82 cell layer for expanding the culture growth. Clonal purity of mutants was assessed by PCR amplification targeting to each genomic region adjacent to the insertion sites and further confirmed by Southern blot analysis of genomic DNA digested with Bgl II restriction enzyme and the resolved fragments hybridized with insertion-specific spectinomycin (*aad*) gene probe [14]. Blots were assessed for the presence of a single predicted DNA fragment for each clonal mutant to define the clonal purity.

### Transcriptional activity of the genes at or near the transposon insertion sites assessed by RT-PCR analysis

Total RNA from *Ehrlichia* cultures (wild-type and mutants), free of contaminated genomic DNA, was isolated as described earlier [14]. Briefly, total RNA was isolated by tri-reagent method (Sigma-Aldrich, St. Louis, MO) and the purified RNAs were treated with RQ1 DNase (Promega, Madison, WI) at 37°C for one hour to remove any genomic DNA contamination. Clearance of genomic DNA was then confirmed by performing real-time RT-PCR assays targeting to 16S rRNA with or without reverse transcriptase as described earlier [14,17]. Specifically, for each set of RT-PCRs, one tube with reverse transcriptase, one tube without reverse transcriptase, one tube using DNA as the template and one tube with no template were included. Concentrations of RNAs from wild-type and clonally purified mutants were then determined by estimating the copy number from the TaqMan real-time RT-PCR data and appropriate dilutions were made to equalize the RNA quantities for all of the RNAs assessed [14,17]. For verifying the RNA expression, the presence or absence of specific product in the assays containing RNA and reverse transcriptase was assessed by comparing the product generated from genomic DNA. Semi-quantitative one-step RT-PCR (Life Technologies, Carlsbad, CA) targeting to *E*. *chaffeensis* genes surrounding the transposon insertion sites was performed with 35 cycles of amplification [18] using the gene specific primer sets (Table 1). The binding locations for the primers within the *E*. *chaffeensis* genome (GenBank # CP000236.1) are provided in Table 1.

**Table 1:** Primers used for RT-PCR of genes surrounding the insertion sites.

Primer name	Gene target	Amplicon size (bp)	Primer sequence (genomic coordinates)
RRG1382	Ech_0202	312	5'-ttg ctg ata gtg tgg cag ctg aag (192727–192746)
RRG1383			5'-tct cca cca tct tgg ata aca gca gg (193038–193013)
RRG1384	Ech_0203	175	5'-tgt gtc ctg ttg tta tgg gtt ctc (194030–194053)
RRG1385			5'-tcc cta agt aat atg gaa cca tct gca c (194204–194177)
RRG1370	Ech_0284	380	5'-tct gct aga agt atg gct act cta gg (267499–267524)
RRG1371			5'- tcc cac agt gta gct ctc tgc (267878–267858)
RRG1372	Ech_0285	409	5'- atg act gct gcc att aca gtt ggg (269137–269160)
RRG1373			5'- cct cat cac cac ttg ttc ctc ctt c (269545–269521)
RRG1632	Ech_0378	447	5'-tgc tat agg gat acc tgt agc ttt tgc (370293–370319)
RRG1633			5'-gca aga cca tcg tac gta cta ggt g (370739–370715)
RRG1276	Ech_0379	374	5'-cta agg ttg tag gga atg caa cc (374161–374183)
RRG1277			5'-aca agg taa gta cct tgc ttg ctc (374534–374511)
RRG1634	Ech_0380	182	5'-atg tgc tct gta tca att gct tg (375004–375026)
RRG1635			5'-aac aaa gaa gta aaa aga cat aca tg (375185–375160)
RRG1374	Ech_0479	357	5'-act cct tgg caa tgg tgt gta g (458414–458435)
RRG1375			5'-aat cgc tct aga caa cac tga agg (458770–458747)
RRG1376	Ech_0480	368	5'-tat gta act tct ttg cct ctt atg (460561–460584)
RRG1377			5'-atg aaa tct tta tta gtg act cga cc (460928–460903)
RRG1636	Ech_0659	225	5'-act aga tga att tga cta tac aat tga tg (672000–672028)
RRG1637			5'-ttt aag ctt tgt aag ctg tta gaa t (672224–672200)
RRG1344	Ech_0660	265	5'-tgt acc tgt atc ctc acc tat cac c (672386–672410)
RRG1345			5'-cta tca att ctt cac ttc cat ttg tgt g (672651–672624)
RRG1638	Ech_0661	156	5'-atc tac tgc tac caa ccc aat ac (673100–673122)
RRG1639			5'-tag tgc ata tgc aat ttc att gtg c (673255–673231)

### Animal infections

Animal experiments with deer and dogs were performed by complying with the Public Health Service (PHS) Policy on the Humane Care and Use of Laboratory Animals, the US Department of Agriculture’s (USDA) Animal Welfare Act & Regulations (9CFR Chapter 1, 2.31), and with approvals of the Oklahoma State University (OSU) and Kansas State University (KSU) Institutional Animal Care and Use Committees (IACUC), and as per the guidelines of the protocols. Laboratory-reared deer and pure-bred laboratory reared beagle dogs were used for conducting infection experiments. Protocols for rearing deer and infection experiments were described in [14]. Deer were obtained from a USDA registered and accredited breeder. About 5–6 month old beagle dogs of either sex were obtained from Covance Research Products (Denver, PA). Both deer and dogs were housed in door at the climate controlled animal facilities at Oklahoma State University and Kansas State University and adlibitum feed and water were provided and all animals were also provided adequate space to freely move around to get their regular exercise/activity. The animals were monitored daily for the health, behavioral changes and twice weekly monitored for body temperature and hematological changes throughout the study. Some animals exhibited mild fever and low platelet count, none of the dogs or deer were clinically ill to the point where they needed additional veterinary care and treatment. Although not needed to use in the study, the humane end point protocols were in place for deer and dogs. A university veterinarian oversaw veterinary care for all of the animals. At the end of each experiment, deer and dogs were euthanized and euthanasia was performed in accordance with the recommendations of the Panel on Euthanasia of the American Veterinary Medical Association.

The animals were injected with transposon mutants as a pool of 8 mutants (two dogs) or a clonally purified mutant *E*. *chaffeensis* (Ech_0284) (one dog). Note; when performing the infection experiments in dogs, we prepared a mutant pool representing all nine mutants; however, we realized soon after infection that the Ech_0284 was missed by error. To represent this mutant in the study, another dog was infected with this mutant. One deer was infected with GFPuv mutant pool containing Ech_0202, Ech_0601, and Ech_0760 [14,17]. Inocula were prepared and inoculated with an estimated concentration of ~2x10^8^ *Ehrlichia* organisms in one ml [14]. Infections with wild-type *E*. *chaffeensis* were also performed several times in dogs and the wild-type pathogen is frequently detected in blood [12,19]. The presence of mutants in an animal was assessed in blood drawn at several days post infection by performing culture recovery and by semi nested PCR analysis [12,14]. Primers for the PCRs are reported previously in Table S1 of Cheng et al. [12,14]. The DNAs were used to assess *E*. *chaffeensis* infection status in the PCR targeting to the insertion specific regions of mutant clones. Briefly, two μl of genomic DNA from deer or dog blood was used for the first round PCRs in a 25 μl reaction volume using Platinum Taq DNA polymerase as per the manufacturer's instructions (Life Technologies, Carlsbad, CA). The PCRs were performed in a GenAmp9700 instrument (Applied Biosystems, Foster City, CA) with the following temperature cycles: 94°C for 4 min, followed by 35 cycles of 94°C for 30 s, 52°C for 30 s, and 72°C for 1 min and 1 cycle of 72°C for 3 min. The second round of PCR was performed using the same PCR conditions as the first round PCR and the templates for the second round included two μl of 1:100 diluted products from the first PCR and with nested PCR primer set. The PCR products were resolved on 1.5% agarose gel [20] and the results were considered as positive (+) or negative (-) depending on the presence or absence of specific size products.

### Needle infection of *A*. *americanum* ticks with mutants or wild-type *E*. *chaffeensis* cultures

*A*. *americanum* nymphal ticks experimentally fed and dropped after completing a blood meal from a naïve sheep were used in this study. The fed nymphs were obtained from the Oklahoma State University tick facility. Within 24–48 h post blood meal, the nymphs were injected with about 5 μl of 10 fold concentrated cultured *E*. *chaffeensis* (mutants or wild-type) containing approximately 5,000 infected cells by following the method described in [21]. Briefly, the *E*. *chaffeensis* infected DH82 culture having 80–90% infectivity was harvested by spinning at 10,000 x g for 10 min and the resulting cell pellet was washed with 1 x phosphate buffered saline (PBS) and was resuspended in 1/10 volume of PBS, which was then used for needle puncture inoculation into the ventral side of ticks. Ticks were then allowed to molt to adults at room temperature [22]. Tick infection experiments were divided into four separate groups. The first group received wild-type *E*. *chaffeensis* infection. The second group received mCherry mutant pool containing five mutants, as reported earlier [14]. The third group received a pool of five clonally purified mutants cultured independently and then mixed to generate approximately equal numbers of all five mutant organisms. The group 4 contained five sub-groups; each sub-group received infections with one each of the five clonally purified mutants. Each group or sub-group of ticks included 100 ticks. Following molting, 14–21 randomly selected adult ticks of both sexes from every group or sub-group were assessed for the infection rates. Total genomic DNA from ticks was individually isolated (Wizard genomic DNA isolation kit, Promega, Madison, WI); resuspended into 100 μl of nuclease-free water and 2 μl each of the DNA was used as template for nested PCR analysis by following the protocol described in the previous section.

## Results

### Clonal purification of *E*. *chaffeensis* mutants by limiting dilution technique

**Fig 1:**
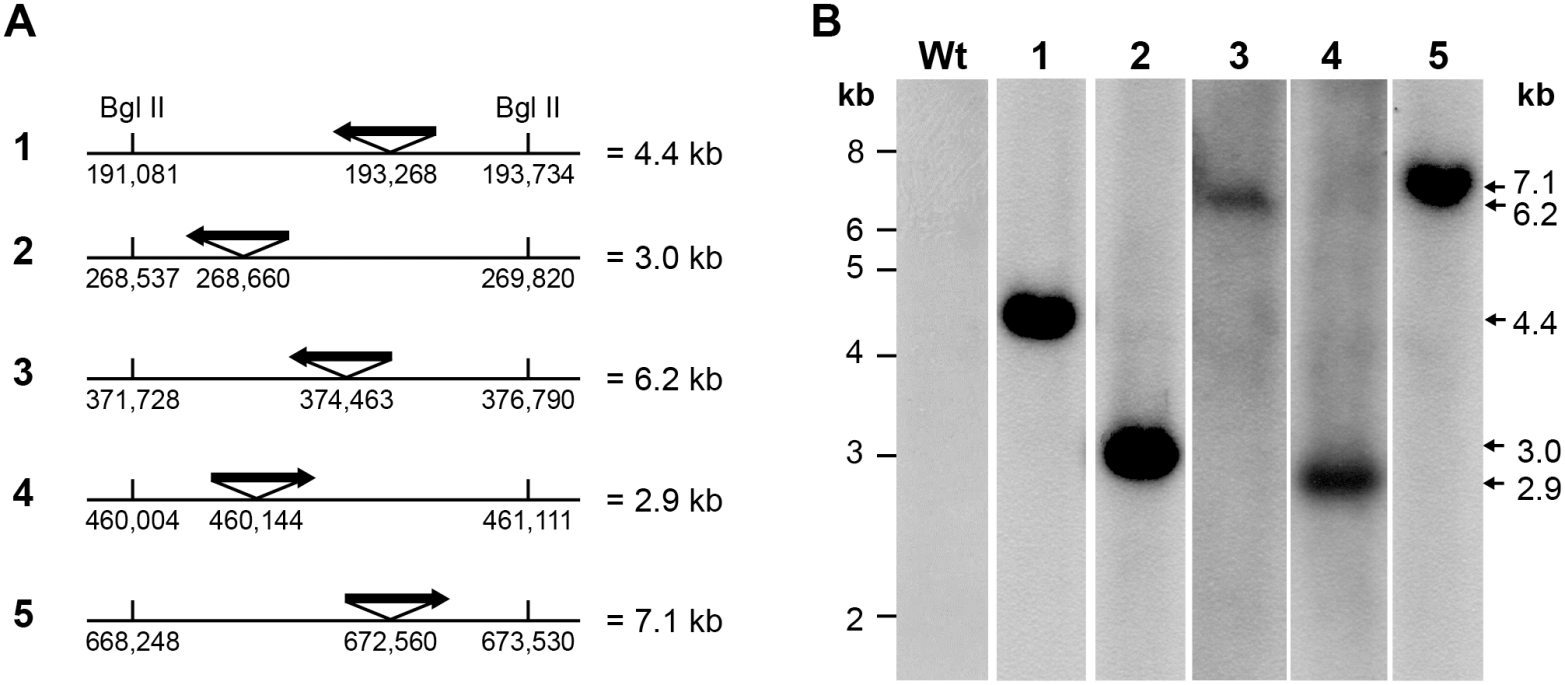
Verification of clonal purity of five *E*. *chaffeensis* mutants by Southern blot analysis. A) Predicted Bgl II digested genomic DNA fragments calculated based on the insertion locations (orientation of the insertions is represented by the arrowheads). B) Southern blot data identifying the expected DNA fragments; lanes Wt and 1–5 represent genomic DNAs derived from *E*. *chaffeensis* wild-type and mutants Ech_0202, Ech_0284, Ech_0379, Ech_0480 and Ech_0660, respectively. Molecular weights of the DNA markers migrated at specific locations were presented at the left end of panel B. Expected size genomic DNA fragments for each mutant were identified on the right end of panel B.

Recently, we reported the establishment and characterization of nine transposon mutants of *E*. *chaffeensis in vitro* and in white-tailed deer [14]. Mutations within Ech_0230, Ech_0379 and Ech_0660 genes causing transcriptional inactivation resulted in the attenuated growth in deer [14]. To further characterize the mutants, we clonally purified five mutants (Ech_0202, Ech_0284, Ech_0379, Ech_0480, and Ech_0660) by limiting dilution technique. Clonal purity of mutants was verified by Southern blot analysis for the five mutants (Fig 1). Expected genomic DNA fragments for the Bgl II restriction endonuclease digestion, estimated from the position of transposon insertions, were calculated for the five mutants (Fig 1A). The predicted fragments were observed in the digested genomic DNA of each purified mutant, but not in wild-type *E*. *chaffeensis* culture derived DNA (Fig 1B). Clonal purity of the mutants was further confirmed by performing PCR analysis targeting to genomic regions adjacent to insertion sites (not shown).

### Impact of mutations on the transcriptional activities of genes near the insertion sites

**Fig 2:**
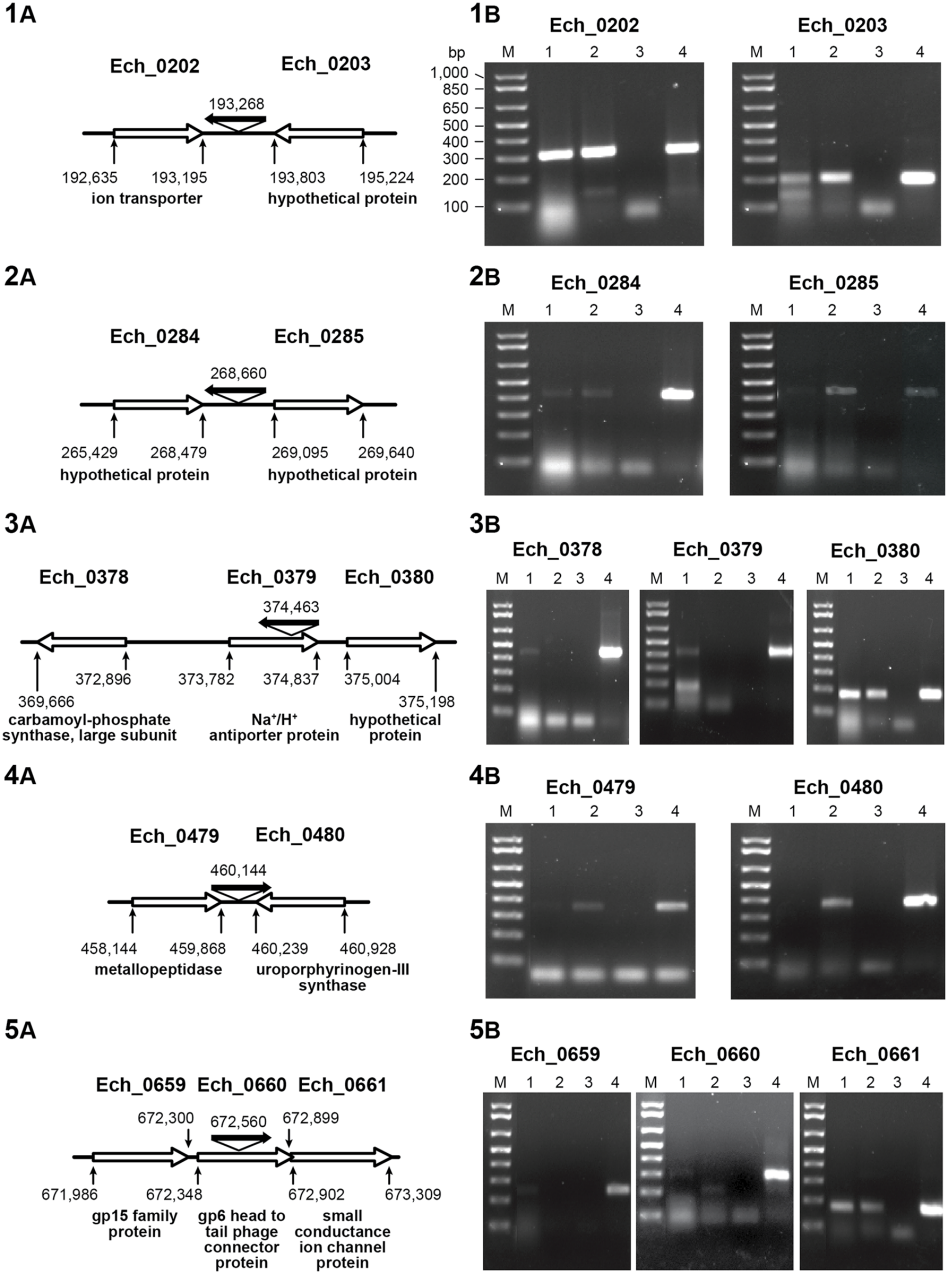
Transcriptional analysis of genes upstream and downstream from five clonal mutants. Genomic DNA-free total RNA from wild-type *E*. *chaffeensis* (lane 1) and clonal mutants (lane 2) was assessed for the genes 5’ and 3’ to insertion sites by semi quantitative RT-PCR. Panels 1A-5A have the cartoons depicting the insertions and their orientations within the genome and the genes near the insertions. The annotated/predicted gene names near the insertions were provided in each sub-panel. Panels 1B-5B had RT-PCR products resolved on agarose gels for each of the respective genes as in panels 1A-5A. No template controls (lane 3) and wild type genomic DNA template reactions (lane 4) were included to serve as negative and positive controls for each reaction, respectively. M represents 1 kb plus DNA ladder (Life Technologies. Carlsbad, CA). Molecular sizes of the resolved DNA ladder shown in the Figs are the same for each sub-panels and were identified in the left image of panel 1B. Predicted amplicon for each gene was listed in Table 1. The primer binding locations relative to whole genome data were also provided in Table 1.

Mutations within Ech_0230, Ech_0379 and Ech_0660 caused transcriptional inactivation from these genes [14]. To assess the polar effects in altering transcriptional activities of genes surrounding the insertions, we evaluated transcription from genes located immediately upstream and downstream to insertion sites for the five clonally purified mutants (Fig 2). All mutations appeared to influence the transcript levels from the genes immediately upstream and/or downstream to the insertion sites. The insertion downstream from Ech_0202 had a minor increase in the transcription from Ech_0203. The mutation 3’ to Ech_0284 caused an enhancement of gene expression from Ech_0285 gene. Similarly, mutation within Ech_0379 caused decline of the transcription to undetectable level from the upstream gene, Ech_0378, similar to the loss of transcription from Ech_0379 gene [14], while not impacting the transcript level for the downstream gene, Ech_0380. The mutation 3’ to Ech_0479 enhanced transcription from this gene and also activated the transcriptionally silent gene, Ech_0480. Mutation within Ech_0660 resulted in the decline in transcription to undetectable level from Ech_0659, similar to transcript knockdown from Ech_0660 gene [14]. This mutation had no impact on the transcript level of Ech_0661.

### Impact of mutations on *E*. *chaffeensis* growth in an incidental host

*E*. *chaffeensis* transposon mutants grow well under *in vitro* culture conditions, while their growth and persistence in the reservoir host, white-tailed deer, is variable [14]; insertions causing transcriptional inactivation from three putative membrane protein encoding genes Ech_0230, Ech_0379 and Ech_0660 resulted in the attenuated growth in deer, while several other mutants and wild-type *E*. *chaffeensis* organisms circulated in blood of the infected animals frequently [12,14]. To examine if the mutations similarly impacted the pathogen’s growth in an incidental host, we conducted experimental infection studies in three dogs; two dogs were infected with 8 mutants and one dog was infected with a clonally purified mutant Ech_0284 (Table 2). As in deer, wild-type *E*. *chaffeensis* in dogs caused persistent infection that is also detected frequently in blood [12]. Infection progression in the dogs was followed for 44 days by sampling blood once every 2–7 days. The dogs tested positive for the mutants similar to our prior observations in deer [14] when assessed by culture recovery and insertion-specific PCRs at various time points post infections (Table 2). As in deer, dogs tested completely negative for the same three insertion mutations at genes Ech_0230, Ech_0379 and Ech_0660, while the others were detected one or more times and at different time points. In addition, the mutant near Ech_0202 gene was also undetectable. Evaluation of Ech_0202, Ech_0601 and Ech_0760 mutants *in vivo* was assessed for the first time. Ech_0601 is an intragenic mutation, while Ech_0202 and Ech_0760 mutations are intergenic mutations downstream from the coding sequences of Ech_0202 and Ech_0760 genes, respectively. As the mutants’ progression in dogs is similar to deer for the previously assessed six mutants, we reasoned that the infection progression with Ech_0202, Ech_0601 and Ech_0760 mutants in deer will also be similar to dogs. To test this hypothesis, we followed infection for two months in a deer with a pool of these three mutants (Table 3). Indeed, Ech_0202 mutant was also undetectable in deer, while Ech_0601 and Ech_0760 mutants persisted.

**Table 2:** Verification of the *E*. *chaffeensis* infection status by nested PCR targeting to the transposon insertion sites in mutant pool infected dog blood.

Days post infection^#^														
	Insertion site	0	2	5	7	9	12	14	16	19	21	29	35	44
Dog 1	Ech_0202	₋	₋	₋	₋	₋	₋	₋	₋	₋	₋	₋	₋	₋
	Ech_0230	₋	₋	₋	₋	₋	₋	₋	₋	₋	₋	₋	₋	₋
	Ech_0379	₋	₋	₋	₋	₋	₋	₋	₋	₋	₋	₋	₋	₋
	Ech_0480	₋	₊	₊	₊	₊	₊	₋	₊	₋	₋	₊	₊	₊
	Ech_0490	₋	₋	₋	₋	₋	₋	₊	₊	₊	₋	₊	₋	₋
	Ech_0601	₋	₋	₋	₋	₋	₋	₋	₋	₋	₋	₋	₋	₊
	Ech_0660	₋	₋	₋	₋	₋	₋	₋	₋	₋	₋	₋	₋	₋
	Ech_0760	₋	₋	₋	₋	₋	₋	₊	₋	₋	₋	₊	₋	₋
Dog 2	Ech_0202	₋	₋	₋	₋	₋	₋	₋	₋	₋	₋	₋	₋	₋
	Ech_0230	₋	₋	₋	₋	₋	₋	₋	₋	₋	₋	₋	₋	₋
	Ech_0379	₋	₋	₋	₋	₋	₋	₋	₋	₋	₋	₋	₋	₋
	Ech_0480	₋	₊	₊	₊	₋	₋	₊	₋	₋	₋	₊	₊	₊
	Ech_0490	₋	₊	₋	₋	₋	₋	₋	₋	₋	₋	₋	₋	₋
	Ech_0601	₋	₋	₋	₋	₊	₋	₋	₋	₋	₋	₋	₋	₋
	Ech_0660	₋	₋	₋	₋	₋	₋	₋	₋	₋	₋	₋	₋	₋
	Ech_0760	₋	₋	₋	₋	₋	₋	₋	₋	₋	₋	₋	₊	₊
Dog 3	Ech_0284	₋	₋	₋	₋	₋	₋	₊	₊	₊	₊	₊	₋	₊

^#^The signs − and + refer to samples tested negative or positive by culture recovery and/or nested PCR, respectively.

**Table 3:** Infection status in deer blood with mutant pool containing Ech_0202, Ech_0601 and Ech_0760.

			Days post infection^#^															
Insertion site	0	5	6	8	10	14	18	21	28	32	35	39	42	46	49	52	59	63
Ech_0202	₋	₋	₋	₋	₋	₋	₋	₋	₋	₋	₋	₋	₋	₋	₋	₋	₋	₋
Ech_0601	₋	₋	₊	₋	₋	₊	₊	₊	₊	₋	₋	₋	₋	₋	₋	₋	₋	₊
Ech_0760	₋	₊	₋	₋	₊	₋	₊	₊	₋	₋	₋	₋	₋	₋	₋	₋	₋	₋

^#^The signs − and + refer to samples tested negative or positive by culture recovery and/or nested PCR, respectively.

### Impact of mutations on *E*. *chaffeensis* growth in the tick vector

The *in vivo* experiments in dogs and deer suggest that disruptions within genes at three *E*. *chaffeensis* genomic locations (Ech_0230, Ech_0379 and Ech_0660) cause attenuated growth (rapid clearance) in deer and dogs. Attenuation was also observed in one intergenic insertion located downstream to Ech_0202 gene coding region. It is possible that the attenuation is the result of disruptions of genes essential for the persistent growth and pathogenesis of *E*. *chaffeensis* only in vertebrate hosts or alternatively, the genes are also essential for the organism’s persistence in its tick vector. We investigated this hypothesis by infecting *A*. *americanum* ticks with *E*. *chaffeensis* mutants by needle inoculation method. In particular, we selected two sets of mutants for this experiment to test this hypothesis; 1) all four attenuated mutants and 2) a sub-set of three mutants that persisted in deer and dogs. Ticks were infected with a pool of mutants or as an individual mutant or with a wild-type *E*. *chaffeensis*. Ticks in group 1 received wild-type *E*. *chaffeensis*, group 2 received the mCherry mutant pool [14] consisting of five mutants (Ech_0230, Ech_0284, Ech_0379, Ech_0480 and Ech_0490), group 3 received an equal mixture of five individually cultured mutants (Ech_0202, Ech_0284, Ech_0379, Ech_0480 and Ech_0660) and group 4 contained five sub-groups; each sub-group was inoculated with one of the five clonally purified mutants as reported in Fig 1 (the same five mutants used in group 3 experiment). Following molting of the needle infected nymphs to adults, total genomic DNA recovered from 14–21 randomly selected ticks of both sexes from each group were assessed for infection rates (Table 4). Infection rate with wild-type *E*. *chaffeensis* (group 1) was 24%. The vertebrate host attenuated mutant, Ech_0379, tested positive in ticks when assessed in a pool or as an individual mutant and the infection rate was 28–30% that is similar to wild-type infection rate in ticks. Similarly, Ech_0230 and Ech_0660 mutants tested positive in ticks and their infection rates are 20% and 7–24%, respectively. As in deer and dogs, Ech_0202 mutant was undetectable in ticks receiving infection as a pool (group 3); however, it was detected in one tick when infected as a clonal mutant. The mutants that persisted in deer and dogs (Ech_0284, Ech_0480 and Ech_0490) also caused infections to ticks.

**Table 4:** Infection rate of needle injected *A*. *americanum* ticks*.

	Group 1 (wild-type)	Group 2 (mCherry pool)	Group 3 (equal mixture of mutants)	Group 4 (individual mutants)
Wild-type	24% (11/46*)			
Ech_0202			0%(0/14)	4.7%(1/21)
Ech_0230		20%(4/20)		
Ech_0284		5%(1/20)	57.1%(8/14)	23.8%(5/21)
Ech_0379		30%(6/20)	28.5%(4/14)	28.5%(6/21)
Ech_0480		50%(10/20)	57.1%(8/14)	57.1%(12/21)
Ech_0490		50%(10/20)		
Ech_0660			7.1%(1/14)	23.8%(5/21)

*tick tested positive/ticks assessed

## Discussion

We reported mutagenesis for the first time for *E*. *chaffeensis* recently [14]. Little is known about how insertion mutations impact the transcriptional activity of genes near the insertion sites. We investigated the polar effects of mutations on transcriptional changes from genes at close proximity to five insertion mutations. This analysis required the use of clonally purified mutants; clonal purification method for intracellular organisms, such as for *Ehrlichia* species, is a very tedious and time-consuming process. We were successful in generating five clonal mutants in this study after several attempts, which took nearly one year to complete. All five mutations altered gene expression from one or both genes located upstream or downstream of the insertions; the changes are independent of the orientation of the genes and the location of an insertion. The mutation downstream to Ech_0284 caused enhanced transcription from the Ech_0285. As the insertion mutation is located upstream to Ech_0285 coding sequence, we reasoned that the insertion influenced the promoter function positively. Interestingly, despite that the insertion is located within the Ech_0379 coding region and about 2 kb downstream from the coding region of Ech_0378; it reduced transcription to undetectable levels from both Ech_0378 and Ech_0379, while causing no change in the 3’ gene (Ech_0380) transcription. It is not clear how the mutation impacted the Ech_0378 transcription as Ech_0378 and Ech_0379 appear to be transcribed from separate promoters; one possibility is that the coding region of Ech_0379 may contain DNA elements controlling Ech_0378 transcription. Alternatively, Ech_0379 gene product may be necessary to activate Ech_0378 promoter. The insertion downstream to Ech_0479 is positioned 3’ to stop codons of both Ech_0479 and Ech_0480 genes and resulted in transcriptional enhancement from both the genes, possibly by enhancing the stability of these gene transcripts.

The genomic analysis surrounding Ech_0660 mutation revealed that the genes Ech_0659, Ech_0660 and Ech_0661 have very little to no intergenic space; the intergenic region between Ech_0659 and Ech_0660 is only 48 base pairs long, while the protein coding regions of Ech_0660 and Ech_0661 overlapped by four nucleotides. The analysis suggests that the three genes belong to an operon. Mutation within the Ech_0660 gene had a very exciting outcome; as predicted for a polycistronic message, transcript levels of Ech_0659 and Ech_0660 genes in wild-type *E*. *chaffeensis* were very similar and Ech_0660 insertion mutation resulted in the complete loss of transcription from both the genes. However, the Ech_0661 transcription was higher in wild-type compared to that of Ech_0659 and Ech_0660 and the mutation did not alter the transcription level of Ech_0661. The data suggest that the gene Ech_0661 is independently transcribed, despite the overlap of its coding region with that of Ech_0660 and the prediction of their presence as an operon. The data also suggest that the promoter for Ech_0661 is within the coding region of Ech_0660. Our prediction analysis revealed the presence of a high AT-rich promoter-like segment upstream to the start codon of Ech_0661 that is within the coding region of Ech_0660 (not shown). There are only a few documented examples of bacterial genomes where part of the coding sequence of a gene serves as the promoter for another gene [23,24]. The predicted presence of a putative promoter and transcriptional differences in genes with overlapping coding regions suggests that the *E*. *chaffeensis* has evolved to efficiently use its reduced genome. Mutations impacting transcriptional changes will allow for detailed investigations to understand the regulation of gene expression of several *E*. *chaffeensis* genes.

Infection assessment of *E*. *chaffeensis* transposon mutants in dogs for 44 days identified the rapid clearance of the same four mutants which cleared rapidly in deer; Ech_0202, Ech_0230, Ech_0379 and Ech_0660. The possibility of competition between mutants *in vivo* leading to over growth of certain mutants, while causing the disappearance of others, cannot be ruled out. We tested this hypothesis recently [19]. Two clonally purified attenuated mutants (Ech_0379 and Ech_0660 mutants) were assessed in deer and dogs and their infection progression was compared with wild-type *E*. *chaffeensis* infection [19]. As observed in infections with pool of mutants, the clonally purified mutants also circulated less frequently, thus arguing against the competition theory.

Interestingly, transposon insertion within the Ech_0601 gene coding region caused transcriptional inactivation *in vitro* [14], but did not impact the mutant’s persistence in dogs or deer. On the contrary, Ech_0202 mutant that had no impact on transcription from this gene and genes upstream and downstream to the mutation was undetectable in deer and dogs when assessed for several weeks. This mutant was also attenuated in ticks, as only one tick tested positive when infected with the purified mutant, but not as mutant pools. These data were puzzling and we reasoned that the mutation impacted transcription from a remote location(s) leading to attenuation. Similarly, other mutations may cause genome-wide changes in the transcription. This hypothesis remains to be tested by performing global transcriptome analysis of mutants.

We adapted the needle infection protocol [21] to generate infected ticks with wild-type and mutant *E*. *chaffeensis* organisms. Numerous infections carried out in the current study to generate infected ticks by needle infection method demonstrated that it is a very valuable tool to generate *E*. *chaffeensis* infected ticks and to assess the impact of various mutations in the pathogen in tick infections. This method will likely be useful for generating infected ticks with other related *Anaplasmataceae* pathogens.

The transposon insertion mutations are stable and represent major insertions, which survive without the need for providing antibiotics to the animals or to the culture media. Recent studies also suggest their value as a useful genetic tool for developing stable introduction of therapeutic transgenes into human cells [25]. All of the mutants we reported in our previous publication [14] are routinely maintained in the absence of antibiotics. Similarly, antibiotics were not fed to the animals or ticks. Likewise, we did not add antibiotics to the culture media when performing culture recovery experiments. Furthermore, nearly four years of continuous *in vitro* culturing of the mutants and the retention of insertions following their circulation in deer and dogs provide evidence that the transposon mutants are stable. These observations are similar to the numerous published studies reporting the generation of stable transposon mutants in several Gram positive and Gram negative organisms [26–32], including the ones reported for two *Anaplasma* species; *A*. *marginale* and *A*. *phagocytophilum* [30–32]. Infections with clonally purified *E*. *chaffeensis* mutants which cleared rapidly in vertebrate animals (mutant clones of Ech_0202, Ech_0230, Ech_0379 and Ech_0660) may offer sufficient protection against wild-type pathogen infection. Indeed, we tested this hypothesis recently by examining two of these mutant clones; Ech_0379 and Ech_0660, and the study suggested that the attenuated mutants are useful in reducing the rickettsemia when challenged with wild-type infections [19].

## Conclusions

We reported that insertion mutations in *E*. *chaffeensis* cause polar effects by altering gene expression from several genes positioned upstream and downstream to the insertion sites. We also reported the differential requirements of functionally active genes in the vertebrate hosts and the tick vector, as assessed by mutagenesis. Further, we presented evidence that mutations at certain *E*. *chaffeensis* genomic locations cause attenuation of the organism in vertebrate hosts, but do not impact its acquisition and persistence in ticks. This study is important in extending future investigations to assess the growth kinetics of mutants and molecular studies to define how gene expression is altered in *E*. *chaffeensis*.
